# Recent Advancements in Topical Anti-Psoriatic Nanostructured Lipid Carrier-Based Drug Delivery

**DOI:** 10.3390/ijms24032978

**Published:** 2023-02-03

**Authors:** Tulshidas S. Patil, Nayan A. Gujarathi, Abhijeet A. Aher, Hemal E. Pachpande, Charu Sharma, Shreesh Ojha, Sameer N. Goyal, Yogeeta O. Agrawal

**Affiliations:** 1 Shri Vile Parle Kelavani Mandal’s Institute of Pharmacy, Dhule 424001, Maharashtra, India; 2 Department of Internal Medicine, College of Medicine and Health Sciences, United Arab Emirates University, Al-Ain P.O. Box 15551, United Arab Emirates; 3 Department of Pharmacology and Therapeutics, College of Medicine and Health Sciences, United Arab Emirates University, Al-Ain P.O. Box 15551, United Arab Emirates

**Keywords:** nanostructured lipid carriers, psoriasis, physicochemical findings, pharmacological findings, regulatory aspects

## Abstract

Psoriasis is linked with unusual differentiation and hyperproliferation of epidermal keratinocytes that significantly impair the quality of life (QoL) of patients. The present treatment options only provide symptomatic relief and are surrounded by various adverse effects. Recently, nanostructured lipid carriers (NLCs) have emerged as next-generation nanocarriers with better physicochemical characteristics. The current manuscript provides background information on psoriasis, its pathophysiology, existing treatment options, and its limitations. It highlights the advantages, rationale, and mechanism of the permeation of NLCs for the treatment of psoriasis. It further gives a detailed account of various NLC nanoformulations for the treatment of psoriasis. In addition, tabular information is provided on the most relevant patents on NLCs for treating psoriasis. Lastly, light is shed on regulatory considerations related to NLC-like nanoformulations. In the treatment of psoriasis, NLCs display a sustained release drug profile, an ability to incorporate both hydrophobic and hydrophilic drugs, an enhancement in skin hydration, penetrability, retention, and bioavailability of the drug, along with reduced staining potential as compared to conventional ointments, and decreased side effects of drug molecules. This affirms the bright future of NLC nanoformulations in the treatment of psoriasis. However, academic industry collaboration and more sound regulatory controls are required to commercialize the NLC nanoformulations for psoriasis treatment.

## 1. Introduction

**Figure 1:**
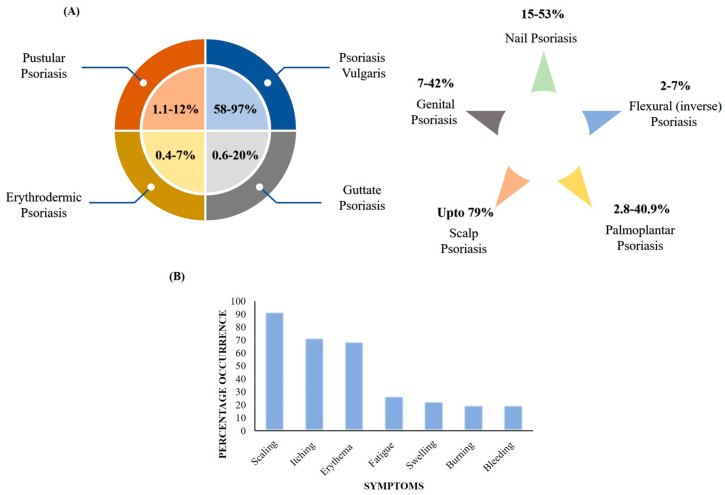
(**A**) Types of psoriasis based on clinical manifestations, anatomical site, and prevalence. (**B**) Common symptoms of psoriasis and percentage occurrence.

Psoriasis is a chronic, noncontagious, excruciating, mutilating, and disabling papulosquamous skin disorder. As per the Global Report on Psoriasis by the World Health Organization (WHO), the overall existence of psoriasis ranges from 0.09% to 11.43% in different countries, in particular, varying from 1.5 to 5% in developed countries. There are a minimum of 100 million people suffering worldwide, which makes psoriasis a severe global issue [1]. In India, the incidence in adults ranges between 0.44 to 2.8% and is more prevalent in males (nearly twice as much) in comparison to females [2]. The disease significantly impairs quality of life (QoL) and further results in high physical, social, and emotional burden. Various types of psoriasis based on clinical manifestation and anatomical site, their percentage prevalence, and common symptoms are depicted in Figure 1A,B. On the basis of clinical manifestations, psoriasis can be categorized as psoriasis vulgaris, guttate psoriasis, erythrodermic psoriasis, or pustular psoriasis. Further, based on the involvement of anatomical region, psoriasis is classified as palmoplantar pustulosis, nail psoriasis, flexural (inverse) psoriasis, scalp psoriasis, or genital psoriasis [3]. The most common and frequent symptoms aligned with psoriasis are scaling, itching, erythema, fatigue, swelling, burning, bleeding, etc. [1].

Psoriasis is a T cell-mediated inflammatory disease mainly caused by an imbalance in inflammatory cytokine release leading to severe epidermal changes. In normal skin, keratinization, i.e., the process of transformation of basal keratinocytes to anucleate corneocytes, takes approximately 50 days, but in psoriatic skin, this process is completed in only 5 days. This leads to shortened epidermal turnover time and the formation of scaly psoriatic plaques on the skin.

The pathophysiology of psoriasis involves the following:

- Keratinocyte hyperproliferation leads to an enhanced buildup of keratin as a thick coat (scaly plaques) in the stratum corneum (SC);
- Dilation and proliferation of dermal blood vessels;
- Accumulation of inflammatory cells and neutrophils and decreased granular tissue or absence thereof [4,5].

**Figure 2:**
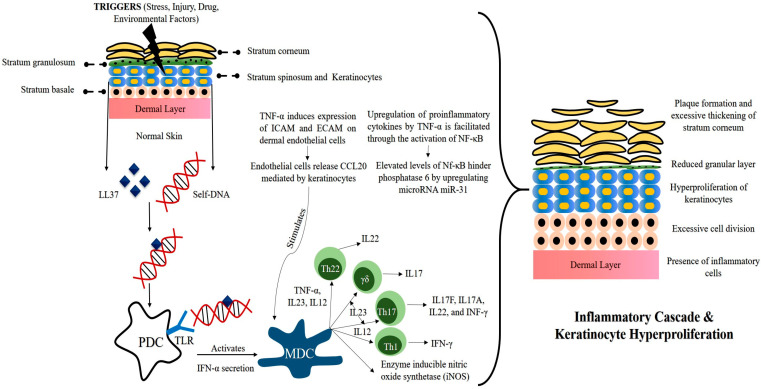
Pathophysiology of psoriasis disease.

The exact sequence of events leading to this aberrant condition has still not been fully elucidated, but some potential triggers and key abnormalities in genetically predisposed persons have been identified. On the basis of the present literature, the pathophysiology of psoriasis disease is depicted in Figure 2.

The inception of psoriasis begins when provocative factors such as trauma, infection, drugs, stress, or physical injury trigger the skin. This leads to the release of psoriatic auto-antigen LL37 (antimicrobial peptide) from keratinocytes. LL37 then forms a complex with self-DNA and activates plasmacytoid dendritic cells (PDCs) by binding to TLR9. PDCs do not normally react to self-nucleotides, but this self-tolerance seems to break in the presence of the LL37 peptide. The activation of PDCs is a potent trigger that leads to a surge of subsequent cytokine storms. Both LL37 and PDCs are overexpressed in psoriatic skin.

Activated PDCs secrete IFN-α, further leading to the activation of myeloid dendritic cells (MDCs). MDCs produce several cytokines such as IL12, IL23, and TNF-α, and under the influence of these interleukins, naïve T-cells differentiate into TH1, TH17, and TH22 cells. These regulatory T-cells then enter the circulation and recruit themselves near the epidermis. Once recruited, they release cytokines such as TNF-α, IFN-α, IL17A, IL17F, IL22, etc. This unopposed release of inflammatory mediators leads to a hyperactive inflammatory cascade and thus abnormal keratinocyte proliferation. Overexpression of several other factors can further facilitate the development of psoriatic plaques. Angiogenic mediators such as vascular endothelial growth (VEGF), tumor necrosis factor (TNF), interleukin (IL)-8, and IL-17 are over-expressed in psoriasis and expedite the abnormality [6,7,8,9,10].

## 2. Present Treatment Options and Limitations

The current treatment options for psoriasis mainly provide symptomatic relief. There are three main lines of treatment, viz., topical therapy, systemic therapy, and phototherapy. Normally, mild psoriasis is treated with topical therapy and followed by phototherapy in the case of inadequate response. Systemic therapy is used for treating moderate to severe psoriasis. To date, there is no treatment that would provide a complete cure for psoriasis. Table 1 depicts the present therapeutic options, the drug candidate used, and their adverse effects [11,12,13,14,15,16,17].

Topical therapy can cause the accumulation of the drug at the target site and show high therapeutic effectiveness with fewer systemic side effects [5]. However, the current conventional topical therapy used for the treatment of psoriasis is surrounded by various limitations, such as deprived permeation and absorption of a drug due to the barrier properties of the skin. Moreover, in psoriasis, epidermal hyperplasia, hyperkeratosis, and reduced hydration of the skin also restrict the drug permeation and ultimately the therapeutic efficacy of conventional topical therapy. Phototherapy is time consuming and expensive from both patients’ and physicians’ perspectives, and only a third of psoriasis patients undergo phototherapy. Meanwhile, systemic therapies with drugs such as Methotrexate and Cyclosporin may harm liver and kidney functions and diminish RBCs, WBCs, and platelet counts [18,19,20].

To overcome these limitations of conventional therapy, novel nanoformulations have been explored in recent decades. Various nanocarriers systems such as polymeric nanocarriers (polymeric nanoparticles, micelles, dendrimers, nanospheres, nanocapsules), vesicular lipid nanocarrier systems (liposomes, niosomes), and particulate systems, e.g., solid lipid nanoparticles (SLNs) and metallic nanocarriers (gold and silver nanoparticles) have been explored for the treatment of psoriasis [21,22,23]. However, these nanocarriers were reported to have various issues, such as poor drug loading, low encapsulation/entrapment efficiency (EE), destabilization during storage, etc.

## 3. Nanostructured Lipid Carriers (NLCs): Advantages, Rationale, and Mode of Permeation for Treating Psoriasis

**Figure 3:**
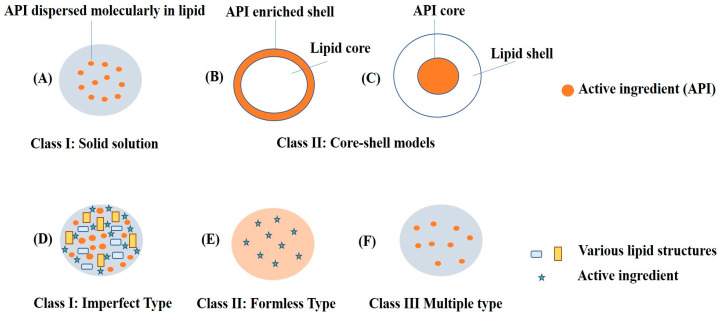
Schematic representation of SLNs: (**A**) solid solution model; (**B**) core-shell model (drug-enriched shell); and (**C**) core-shell model (lipid-enriched shell) and NLCs: (**D**) imperfect type of drug encapsulated in a gap between fatty acid chain of solid and liquid lipids; (**E**) formless-type mixture of solid and liquid lipids preventing crystal formation; (**F**) multiple-type nanocompartments of oils containing dissolved drug.

**Figure 4:**
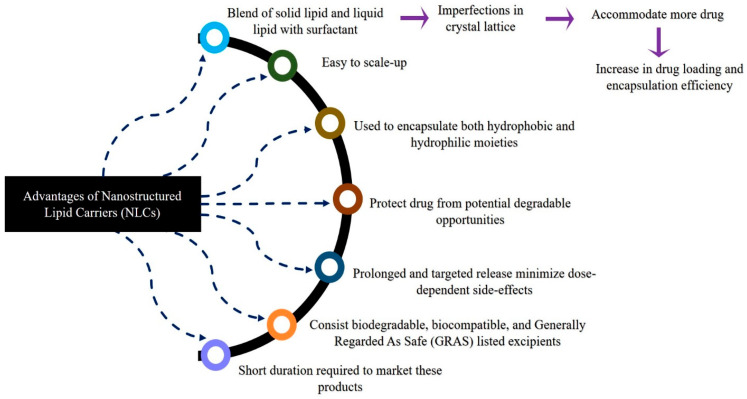
Advantages of nanostructured lipid carriers (NLCs) over other nanocarriers.

NLCs are next-generation solid lipid nanoparticles in which a combination of liquid lipids and solid lipids are used, which creates a disordered matrix, hinders the recrystallization process, and allows for more space for drug accommodation. SLNs are comprised of approximately 0.1–30% *w*/*w* of solid lipids, which is dispersed in an aqueous phase. Meanwhile, in NLCs, a portion of the solid lipid is replaced with a liquid lipid, and a blend of solid and liquid lipids is utilized in a ratio that ranges from 70:30 to 99.9:0.1. Surfactants are used in concentration range of 0.5 to 5% to enhance stability. Figure 3 depicts schematic representations of SLNs and NLCs. Further, Figure 4 depicts the various advantages of NLCs over other nanocarriers such as SLNs, liposomes, and polymeric nanoparticles [24,25,26,27,28].

**Figure 5:**
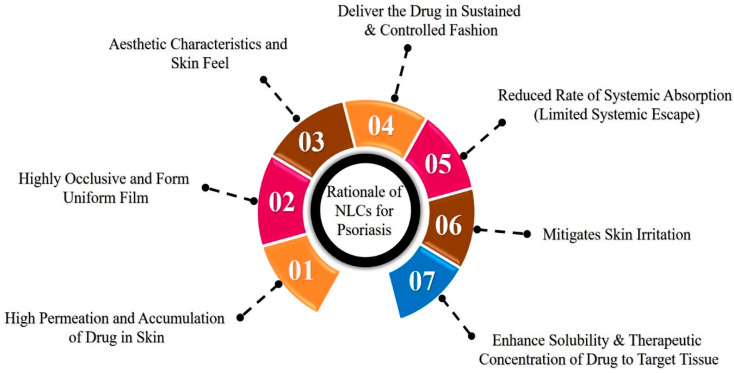
Rationale behind the selection of NLCs for the treatment of psoriasis disease.

The rationale behind the selection of NLCs for the treatment of psoriasis is depicted in Figure 5 and discussed in the subsequent text. From NLCs, a high quantity of a drug can be accumulated and permeates into the skin due to the presence of lipid constituents, the permeation-enhancing effect of surfactants, and small particle size (PS).

Lipid constituents used in the fabrication of NLCs are more analogous to SC lipids, and their interaction with SC lipids promotes the deposition effect by creating a reservoir that ultimately leads to a prolonged residence time of the drug in the skin. Further, liquid lipids such as oleic acid have the ability to integrate with skin lipids and disturb their lamellar arrangements, thereby contributing to a greater drug retention and penetration effect [29]. Surfactants present in the NLCs act as permeation enhancers by extracting skin lipids and enhancing membrane fluidization. The lower PS (<200 nm) of NLCs is targeted to ensure greater surface area and close interaction with the stratum corneum, which enhances the permeation of the drug. NLCs have a highly occlusive nature, and they adhere to the skin absolutely due to their small size and higher lipid content. Consequently, an even film is formed onto the SC. The opaque nature of NLC formulations, the fluidity due to the presence of oil lipids, and the absence of thickening agents provide an aesthetic appearance and skin feel [30,31,32].

NLCs enhance the solubility and therapeutic concentration of drugs in targeted tissues with restricted systemic escape. The delivery of lipophilic drugs can be explained by various mechanisms, as described by Pradhan et al. and Kilfoyle et al. First, the enhanced solubility of the drug in lipid constituents and in turn increases the concentration gradient at the skin surface. The lipoid milieu of the stratum corneum allows for the lipophilic drug to remain in its intercellular portion as a depot and allows for its slower permeation in the lower epidermal layers where psoriasis begins. Then, the increased hydrophilic nature of the dermis layer confines the partitioning of the lipophilic drug. Furthermore, the permeability of psoriatic skin is greater than normal skin and thereby shows more deposition of lipophilic drugs in the epidermis [18,33].

Lin et al. have researched the delivery of hydrophilic drugs such as methotrexate from NLCs. Researchers suggest that surfactants and the saturated and unsaturated fatty acid derivatives used as a source of lipids are responsible for reducing the functional barrier of SC and act as a penetration enhancer for hydrophilic moieties [34].

**Figure 6:**
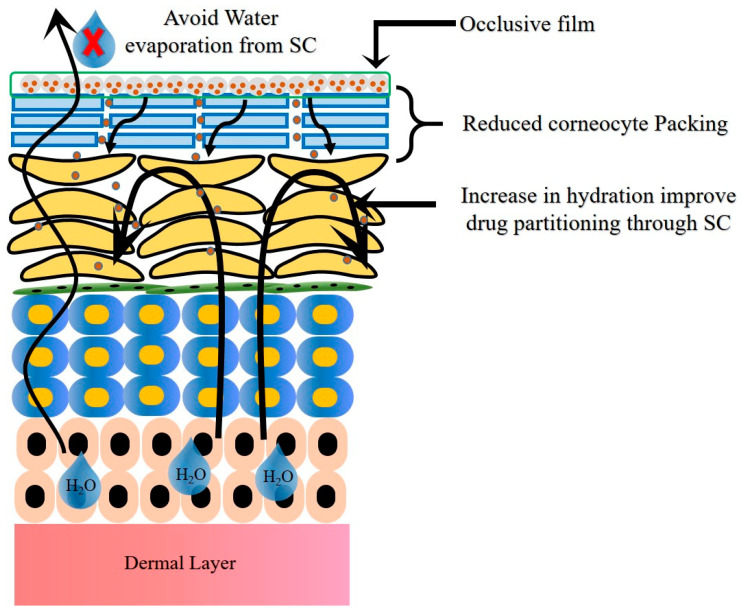
Mode of permeation from NLCs through psoriatic skin.

The mode of permeation from the NLCs through psoriatic skin is depicted in Figure 6. The occlusive nature and reduced PS of NLCs enhance the adhesion of NLCs to the skin. This leads to reduced packing of the keratinocytes due to hydration of the SC and widens the intercellular bilayers, which facilitate the drug permeation into deeper layers. The lipid matrix present in the NLCs exhibits high imperfections in the crystal lattice and modulates the drug release and delivers the drug in a sustained and controlled fashion. Lastly, NLCs mitigate skin irritation due to the use of biocompatible ingredients [35,36].

## 4. Selection of Excipients in Fabrication of SLNs and NLCs and Their Roles

A cautious selection of excipients, such as solid lipids, liquid lipids, and surfactants, and their levels in the fabrication of NLCs have a pivotal role in controlling the PS, encapsulation efficiency (EE), release profile, and retention into the skin with minimal systemic escape. Various solid lipids, liquid lipids, surfactants, other excipients, and solvents used in the formulation of NLCs against psoriasis treatment are listed in Table 2.

The preparation of NLCs with the appropriate lipids helps in enhancing stability, reducing PS, increasing encapsulation, and improving permeation. To take an example, Arora et al. in their work revealed that the selection of glyceryl monostearate (GMS) as a solid lipid not only has greater lipid solubility but also exhibits a self-emulsifying property. Thus, it assists the surfactants in decreasing the PS of the NLCs [30]. Lin et al. in their work demonstrated that the free fatty acids resulting from the solid lipids Precirol and Myverol in the NLCs served as skin penetration enhancers and promoted the permeation of the calcipotriol drug. The selection of a suitable liquid lipid (oil) and its concentration increases the imperfections in the crystal lattice and thereby improves the solubility and overall payload of the drug. Further, due to the presence of oil, the partial or complete eviction of drugs that occurs due to the solidification of solid lipids at room temperature would occur to a minor extent. Solubility screening studies of drugs in solid and liquid lipids act as a crucial step and an important prerequisite in the fabrication of NLCs [34].

Further, the appropriate selection of surfactants directly affects the PS and polydispersity index (PDI). The concept of required HLB (rHLB) value is applied by utilizing a combination of surfactants to attain the NLCs’ formulations with the minimum PS and PDI. The optimized levels of the blend of surfactants contribute to the stabilization of NLCs by avoiding the aggregation of the greatly unstable surfaces [5]. Additionally, each solid and liquid lipid has its inherent rHLB that, if attained, can result in proper emulsification of the lipid phase to achieve a smaller size.

## 5. Detailed Account of NLCs for Treating Psoriasis

### 5.1. Comparative Assessment Studies of NLCs with Other Nano-Lipoidal Carriers

Tretinoin (TRT) is an all-trans-retinoic acid that is commonly used for various skin problems such as acne, psoriasis, skin cancer, and photoaging. It regulates various cellular processes, such as the growth and differentiation of epithelial cells, surface modifications, immune modulation, collagen synthesis, and sebum production. However, its topical preparation is linked with various adverse effects, such as erythema, peeling, and skin irritation, which leads to poor patient compliance. Further, it carries physicochemical limitations such as high lipophilicity and great instability in presence of light, heat, and air. To overcome these limitations, Raza et al. carried out a comparative evaluation of lipidic nanoparticle system SLNs and NLCs with vesicular system liposomes and ethosomes of tretinoin for the treatment of psoriasis [37].

In preliminary studies, researchers reported a mean PS of 182 nm, 120 nm, 82.3 nm, and 79.5 nm; a zeta-potential of 0.67 mV, −15.6 mV, −20.1 mV, and −23.5 mV; and EEs of 65.01%, 76.42%, 86.25%, and 92.13% for liposomes, ethosomes, SLNs, and NLCs, respectively.

Thus, the highest EE of TRT was reported for NLCs, which was justified due to the presence of liquid lipid isopropyl myristate. Transmission electron microscopic (TEM) studies depicted the spherical-shaped morphologies of the prepared nano-lipoidal carriers. These carrier systems were further incorporated into bioadhesive hydrogels and assessed for various in vitro, ex vivo, and in vivo parameters. In doing this, researchers noticed higher photostability, skin penetration, and anti-psoriatic activity for SLNs and NLCs as compared to liposomes, ethosomes, and the marketed formulation. Nevertheless, all the nano-lipoidal formulations were observed to have enhanced biocompatibility and efficacy in comparison to the marketed formulation. In concluding remarks, researchers have stated that for treating superficial skin diseases such as psoriasis, liposomes were found to be as efficient as SLNs and NLCs. Meanwhile, to improvise the photostability of tretinoin-like drugs, SLNs and NLCs can be a superior choice as compared to liposomes and ethosomes [37].

Agrawal et al. have performed a comparative assessment of capsaicin-loaded SLNs (CAP-SLNs) and CAP-NLCs for the treatment of psoriasis. CAP-SLNs and CAP-NLCs were prepared by solvent diffusion method. In the preliminary characterization, CAP-NLCs showed greater EE (87.4%) as compared to CAP-SLNs (79.7%). TEM studies showed the nanometric size range of both the SLNs and NLCs. The permeation of CAP from SLNs and NLCs was checked on the albino rat’s skin. CAP-NLCs and SLNs showed nearly two-and-a-half and one-and-a-half greater permeation flux than plain CAP solution. Further, the in vivo drug localization of the SLN and NLC formulations in different layers of the skin was demonstrated in albino rats. CAP was depicted to have 4.48-fold and 3.13-fold greater retention with NLC and SLN formulations in comparison to its plain solution. Therefore, researchers have reported NLCs to be a more effective carrier system for localized delivery of CAP against psoriasis disease [29].

Tripathi et al. compared the potential of methotrexate-loaded SLN and NLC carbomer gels for treating psoriasis disease. MTX-SLNs and MTX-NLCs were prepared by solvent diffusion method. NLC formulations showed PS ~221 nm due to an increased level of oleic acid, but it was not significantly higher than that observed in SLNs, i.e., 212 nm. Meanwhile, the greater concentration of oleic acid created an imperfection in the structure of the NLCs and ultimately a two-fold greater EE ~62.72% as compared to 26.84% observed in SLNs. In scanning electron microscopy (SEM) studies, SLNs were depicted as ovoid to nearly spherical, NLCs showed nearly spherical-shaped morphologies, and both carriers were observed to have smooth surfaces [39].

In skin permeation studies performed on Wistar Stain albino rats, both the MTX-NLC and MTX-SLN hydrogels depicted extended drug release up to 24 h. MTX-NLC hydrogels showed a higher percentage of drug deposition of ~28.8% as compared to MTX-SLN hydrogels (18.6%). The fluorescence microscopic studies suggested the better localization effect of both lipid nanoformulations on deeper skin regions. Further, the Draize-patch test indicated considerably less irritation as compared to plain MTX-hydrogels. Here, researchers hypothesized that the incorporation of MTX in SLNs and NLCs has reduced the interaction of the acidic functional group (-COOH) of MTX (that triggers the erythematic events) with the stratum corneum and thereby diminished erythematic episodes. In storage stability studies, MTX-SLNs depicted a rise in PS as compared to MTX-NLCs, which indicated the conversion of SLNs into a more structured crystalline lattice and drug leakage during storage [39].

### 5.2. Dual Drug-Loaded NLCs for Psoriasis Treatment

A combination therapy would be beneficial for the effective control of psoriasis disease. However, inconvenience in administration is the main hurdle in combination therapies. The incorporation of both active ingredients in a single drug delivery system has proven to show better patient compliance. NLCs have emerged as a latest-generation lipid-in-water nanoparticulate system and a carrier that can incorporate two drugs with extreme polarities.

Calcipotriol (CAL) is a synthetic vitamin D3 analog utilized for the treatment of psoriasis. However, it leads to cutaneous irritation in nearly 20% of patients. Therefore, efforts have been made to combine CAL with other drugs that may reduce the required dose of CAL and thereby minimize skin irritation [34].

Lin et al. developed calcipotriol (log P = 4.6)- and methotrexate (log P = −1.85)-loaded NLCs (CAL-MTX-NLCs) for the management of psoriasis. CAL-MTX-NLCs were formulated by rotary evaporation and a probe sonication method that contained 0.06% CAL and 0.08% MTX encapsulated in the lipid and aqueous phases, respectively. CAL-MTX-NLCs depicted a PS range of 267–320 nm, PDI ~0.300, and zeta-potential (ZP) from −42 to −45 mV. In in vitro studies, CAL-MTX-NLCs formulations showed reduced release and permeation of CAL after the incorporation of MTX, whereas, MTX showed effective release and 2.4–4.4 times higher permeation using NLCs as compared to the control group. Further, in vivo confocal laser scanning microscopy (CLSM) studies exhibited a good correlation with the in vitro results obtained for the skin targeting of NLCs [34].

Arora et al. also performed a comparative evaluation of cyclosporin and calcipotriol dual-drug loaded SLNs and NLCs for the treatment of psoriasis. In a preliminary evaluation, both types of nanocarriers showed ultra-nano size (<100 nm), high EE, and spherical-shaped morphology. Further, SLN and NLC carriers were poured in Carbopol^®®^934P NF gel and evaluated for various in vitro, ex vivo, and in vivo efficacy parameters. In in vitro studies on HaCaT cell lines, the NLC gel showed greater cellular uptake, with the highest cell viability reduction. In ex vivo dermatokinetic studies, both the lipid formulations depicted the deep and confined permeation of drugs in epidermal layers in comparison to free drug treatment. Further, in in vivo anti-psoriatic efficacy studies in BALB/c mice, groups applied with the NLC gel depicted a maximum decrease in skin inflammation, with no scaly lesions on the skin surface, versus those treated with SLN gel and marketed Betagel preparation [30].

Viegas et al. formulated multifunctional NLCs for the co-delivery of Tacrolimus (TAC) and SiRNA to treat psoriasis disease. The developed NLCs showed PS ~230 nm, PDI 0.390, ZP −17.8 mV, and a high EE of 93.13%. Release and permeation studies depicted controlled release and high skin retention. The viability and uptake studies performed on murine fibroblast cells showed less toxicity with the uptake of NLC in 4 h. Further, in vivo animal studies confirmed the efficacy of the NLCs by offering a synergic effect of TAC and siRNA and a seven-fold decrease in TNF-α expression [40].

### 5.3. Single Drug-Loaded NLCs for Psoriasis Treatment

#### 5.3.1. Acitretin

Acitretin (ACT; 13 cis–trans-retinoic acid) is a metabolite of vitamin A and has various physiological effects, such as regulating the growth and proliferation of epithelial cells and the production of sebum and collagen. Presently, acitretin via the oral route is prescribed for treating severe psoriasis in adults. However, its usage is restricted due to systemic adverse effects and teratogenicity. On the other hand, its topical administration is limited due to its various drawbacks, such as skin irritation, less aqueous solubility, and greater environmental instability.

The skin irritation and burning further lead to a decrease in the acceptability of patients [19]. To minimize these limitations, Agrawal et al. explored the effectiveness of NLCs for enhancing the topical delivery of ACT in the treatment of psoriasis disease.

Initially, acitretin-based NLCs (ACT-NLCs) were fabricated by a solvent diffusion method and optimized by applying a 32-full factorial design (FFD). The optimized ACT-NLCs showed a spherical shape morphology with a PS of 223 nm, a ZP of −26.4 mV, and an EE of 63.0%. In DSC, the absence of an endothermic peak, and in X-ray powder diffraction (XRD), the deformed peak of the ACT drug depicted its transition into an amorphous state and uniform dispersion in NLCs. ACT-NLCs were poured in carbopol 934 P gel (1% *w*/*w*). In in vitro skin deposition studies on human cadaver skin (HCS), researchers demonstrated significantly greater deposition of about 81.38% from ACT-NLC gel versus 47.28% from the plain ACT gel. Further, the clinical effectiveness of the ACT-NLC gel was evaluated on twelve psoriasis patients over a four-week duration. In these studies, researchers found significant progress in therapeutic response and reduced local side effects. Thus, researchers successfully demonstrated the potential of the ACT-NLC gel for the treatment of psoriasis. [19].

#### 5.3.2. Methotrexate (MTX)

Methotrexate (MTX) is primarily a folic acid antagonist and antineoplastic agent that is used effectively in the management of psoriasis. It has been reported that MTX decreases the mitotic activity of psoriatic epidermal cells by selectively inhibiting their DNA synthesis [39]. However, adverse effects such as nausea and vomiting, greater inter-individual variability, and lower bioavailability have limited its oral use. After subcutaneous or intramuscular injection, it precipitates into hepatotoxicity. Further, its topical use is associated with poor permeation through the skin because of its hydro-solubility and greater molecular weight, i.e., 454.56 g/mol [31].

Therefore, to enhance the efficacy of MTX, Pinto et al. formulated methotrexate (MTX)-loaded NLCs (MTX-NLCs) as a new topical therapy for psoriasis. MTX-NLCs were developed by the newly modified hot homogenization and ultrasonication method by utilizing either polysorbate 60 (MTX-NLCs-P60) or polysorbate 80 (MTX-NLCs-P80) as surfactants. The NLCs (placebo and MTX-loaded) showed a PS between 274–298 nm, a lower PDI (<0.25), a ZP nearly −40 mV, and spherical-shaped morphology in SEM studies. The NLC formulations were found to be stable at 25 °C for 28 days. In vitro drug release studies at pH 7.4 showed faster drug release of 64% in the initial 2 h, followed by extended release over 8 h. Further, in in vitro skin permeation studies on pig ear skin, MTX-NLCs-P60 showed higher skin penetration of 5.8% and greater flux of 0.88 µg/cm^2^/h as compared to 4.2% and 0.66 µg/cm^2^/h for MTX-NLCs-P80. Thus, researchers proved the efficiency of NLCs to deliver MTX topically in the treatment of psoriasis [31].

Avasatthi et al. fabricated a MTX-NLC-based nanogel for the management of psoriasis. MTX-NLCs were prepared by the hot homogenization technique and optimized by 33-Box–Behnken design (BBD). Initially, MTX-NLCs depicted a PS of 278 nm, PDI of 0.231, and EE of 22.29%. Then, a MTX-NLC-based nanogel was prepared using carbopol (0.25% *w*/*w*) and ethylene diamine tetraacetic acid (0.05% *w*/*w*). The MTX-NLC gel showed slower and prolonged drug release of ~47.32% after 48 h. Further, in anti-psoriatic activity studies, the MTX-NLC gel showed a substantial decrease in the Psoriatic Area and Severity Index (PASI) score with the retrieval of the mice’s skin. Thus, researchers claimed MTX-NLC gel as an encouraging option to the present MTX formulation for treating psoriasis disease [41].

Agrawal et al. formulated a MTX-NLC topical gel and evaluated its role against psoriasis disease. MTX-NLCs were formulated using a solvent diffusion method and optimized by applying 32-FFD. The nanocarriers were evaluated for various physicochemical parameters. The MTX-NLCs were further added into 1% *w*/*w* carbopol 934 P gel and evaluated for in vitro skin deposition studies in HCS and in vivo anti-psoriatic activitan y in Balb/c mice. The developed MTX-NLCs showed a PS ~253 nm, a ZP −26.4 mV, and EE of 54%. In TEM studies, a rod-shaped morphology of the NLCs was observed. Differential scanning colorimetric (DSC) and XRD studies indicated the amorphous state of the drug within the nanocarrier system. The MTX-NLC formulations showed a slow and sustained release profile. In HCS, the MTX-NLC gel showed higher drug deposition (71.52%) as compared to a plain MTX gel. Moreover, in the in vivo imiquimod (IMQ)-induced psoriatic mouse model, MTX-NLCs showed a reduction in the psoriasis area and other pharmacological and histopathological alterations [20].

Thus, the researchers successfully demonstrated the potential of NLC-based formulations in decreasing the side effects of MTX and enhancing patient compliance for the treatment of psoriasis.

#### 5.3.3. Fluocinolone Acetonide (FA)

FA is a synthetic hydrocortisone derivative, mainly utilized to diminish skin inflammation in managing psoriasis disease. However, its use has been limited due to its dose-dependent adverse effects such as reduced pigmentation, dermal atrophy, acne, and allergic contact dermatitis.

Pradhan et al. developed fluocinolone acetonide NLCs (FA-NLCs) as a topical formulation against psoriasis disease. FA-NLCs were formulated by a microemulsion technique and optimized using 33-BBD. In a preliminary evaluation, the FA-NLCs showed PS 153.54 nm, PDI 0.01, ZP −36.20 mV, drug EE 92.29%, drug loading 2.84%, and sustained drug release of 66.91% after 24 h. TEM studies depicted spherical or oval-shaped morphology and non-aggregated particles with narrow distribution. DSC and XRD analysis showed the transition of the drug from its crystalline state to an amorphous state after incorporation into NLCs. The FA-NLCs were found to be stable over three months at 4°C. Further, in in vitro deposition studies in goat skin, the FA-NLCs showed significant accumulation in the epidermal and dermal layers of skin. Thus, researchers postulated that the selective retention of the FA by NLCs in the epidermal layer may eradicate its adverse effects associated with systemic exposure [18].

#### 5.3.4. Curcumin

Curcumin has been traditionally used as a medicinal plant. It has proven its efficacy in reducing the inflammation in psoriasis by reducing free radicals via different mechanisms such as the suppression of the NF-κB, IL-1β downregulation IL-6, and TNF-α. However, its conventional formulations depict limited bioavailability after oral administration due to its minimal aqueous solubility, low stability, and extensive first-pass metabolism. To curb this, Rapalli et al. formulated curcumin-loaded NLCs (CUR-NLCs) for treating psoriasis and microbial-mediated acne vulgaris. NLCs were formulated by the hot emulsification and probe sonication method and characterized for various in vitro and ex vivo parameters. The developed NLCs showed average PS 96.2 nm, EE 70.5%, and ZP −15.2 mV, and a sustained drug release profile over 48 h. In ex vivo permeation studies performed on goat ear skin, the CUR-NLC-incorporated gel showed 3.24-fold enhanced skin permeation and retention as compared to plain CUR-loaded gel. Further, in cell line studies performed on keratinocyte cells, the CUR-NLC-incorporated gel depicted no signs of toxicity and enhanced cellular uptake [5].

Kesharwani et al. also developed CUR-NLCs for the topical treatment of psoriasis disease. CUR-NLCs were fabricated by a high-speed homogenization method and optimized using 33-BBD. The developed NLCs showed a controlled PS of 189.4 nm, a low PDI of 0.262, a ZP of −21.45 mV, and a high EE of 86.72%. SEM and atomic force microscopy studies confirmed the smooth spherical-shaped morphology of the NLCs. NLCs depicted a sustained release pattern with 60.2% drug release in 24 h. Ex vivo permeation studies showed a decent permeation flux of 0.453 µg/cm^2^/h and 60.2% retention of CUR in the skin epidermis layer. Thus, both of these research investigations marked the potential of NLC-based formulations to enhance the efficiency of CUR for the effective treatment of psoriasis. However, these research studies lacked in vivo anti-psoriatic activity [42].

Kang et al. formulated a cellulose nanofiber film incorporated with CUR-NLCs (CUR-NLCs-CN) against psoriasis disease. NLCs were formulated by a solvent diffusion method with a mean PS of ~500 nm. SEM studies of the CUR-NLCs-CN films revealed a smooth surface as compared to blank films. These observations were justified by the fusion of NLCs with CNF film. The lipid matrix of the NLCs showed a permeation-enhancing effect and the transition of CUR to its amorphous state, which was confirmed by DSC and XRD studies. In IMQ-induced psoriatic mice, groups treated with the CUR-NLCs-CN films showed greater than two-fold enhancement in CUR deposition in the epidermal skin layer as compared with the films deprived of lipids. Further, the CUR-NLCs-CN films showed a decrease in the pro-inflammatory cytokine levels comparable to marketed topical corticosteroid cream [44].

#### 5.3.5. Apremilast (APM)

Apremilast (APM) is a BCS class IV drug that varies greatly in its oral bioavailability. Additionally, its conventional products have dose regimens and tolerability problems that have led to patient incompliance.

Madan et al. fabricated apremilast-containing NLCs (APM-NLCs) for treating psoriasis. Initially, APM-NLCs were formulated by the cold homogenization method by applying 32- FFD. The APM-NLCs were further incorporated into carbopol 940-based hydrogels for topical application. The optimized NLCs showed PS 758 nm, PDI 0.339, EE 85.5%, and ZP −33.3 mV. SEM studies revealed the spherical shape of the NLCs. Reduced crystallinity of the APM due to incorporation in NLCs was observed in XRD studies. The APM-NLCs were found to be stable over three months, and no phase separation was noticed. Further, the APM-NLC hydrogel showed slower diffusion, 48.72% permeation, and 60.1% skin deposition. Thus, researchers demonstrated the potential of NLCs in delivering the poor water-soluble drug with improved drug deposition in the skin for treating psoriasis disease [38].

#### 5.3.6. Pentoxifylline (PTX)

Pentoxifylline (PTX) is an anti-inflammatory drug that is clinically advised in the treatment of psoriasis. However, the hydrophilic nature of the drug prevents it from concentrating on the skin layers, which limits its permeation in the psoriatic lesions. To overcome this, Ghate et al. developed pentoxifylline-loaded NLCs (PTX-NLCs) for the treatment of psoriasis. The PTX-NLCs were formulated by a novel microwave irradiation-assisted thin film process. The prepared NLCs were characterized for various in vitro parameters and ex vivo skin permeation studies. Further, the efficiency of the PTX-NLCs was demonstrated in Swiss albino mice against IMQ-induced psoriasis. The developed PTX-NLCs showed PS < 200 nm, PDI < 0.250, ZP < −28 mV, drug loading of 10%, and EE of 90%. In permeation studies, the NLCs showed a permeation flux of 4.84 μg/cm^2^/h at the end of 24 h, which indicated greater retention of PTX within the skin. In in vivo efficacy studies, the histological examinations showed a significant reduction in the epidermal thickening and the keratin deposition on the skin surface. Thus, researchers proved the ability of NLCs as a promising topical delivery system for PTX against psoriasis disease [4].

#### 5.3.7. Fucoxanthin (FUCO)

Fucoxanthin (FUCO), a marine carotenoid, shows anti-proliferative properties and can be used to treat hyper-proliferative conditions such as psoriasis disease. Cordenonsi et al. fabricated FUCO-loaded NLCs (FUCO-NLCs) and coated them with chitosan (CS-FUCO-NLCs) to enhance the mucoadhesion and wound-healing properties. Initially, NLCs were formulated by the high shear homogenization technique and evaluated for in vitro physicochemical properties, cell viability, and cellular uptake studies on normal dermal human fibroblast cells. The pharmacological assessment was performed in a psoriatic-like cellular model. Further, the antiproliferative effect of the CS-FUCO-NLCs was determined by studying their influence on the expression of the anti-apoptotic gene Bcl-2 using a real-time PCR technique. FUCO-NLCs and CS-FUCO-NLCs showed an increase in the PS of 297.1 nm and 416.3 nm as compared to 253.7 nm of blank NLCs. The CS-FUCO-NLCs showed less uniform size in TEM analysis, positive ZP, and decent bioadhesion properties. The CS-FUCO-NLCs depicted greater uptake in fibroblasts. Moreover, proliferation studies demonstrated no toxic effect and statistically reduced expression of Bcl-2 after incubation with CS-FUCO-NLCs as compared to untreated psoriatic-like cells [45].

#### 5.3.8. Dithranol (DTN)

Dithranol is one of the most effective anti-psoriatic drugs that inhibits keratinocyte proliferation. Though it is harmless and efficacious, its use is upsetting because of its irritating and necrotizing effect on normal and diseased skin. DTN converts into danthron dimer and danthron. Danthron is ineffective against psoriasis, and the dimer form has a staining ability. To overcome these limitations, various conventional formulations such as creams, gels, and ointments as well as novel carriers such as liposomes, niosomes, and dendritic carriers were investigated and are also available on the market [43]. In one of the recent studies, Sathe et al. developed a DTN-NLCs gel and compared its effectiveness with dithranol ointment. The developed NLCs showed PS < 300 nm, PDI < 0.3, and EE of nearly 100%. The DTN-NLC gel showed deep penetration and a sustained release drug profile. Further, in the IMQ-induced psoriatic plaque model, the DTN-NLC gel showed a substantial decrease in the erythema, skin thickness, and scaling as compared to the negative control. Additionally, the DTN-NLC gel showed much less staining potential as compared to ointment. Thus, the DTN-NLC gel was found to be a more effective and acceptable treatment option versus the available ointment for treating psoriasis disease [43].

#### 5.3.9. Mometasone Furoate (MF)

Mometasone furoate (MF), a prodrug of mometasone, is a non-fluorinated synthetic corticosteroid primarily utilized topically to decrease skin inflammation in psoriasis and eczema. It possesses anti-inflammatory, anti-pruritic, and anti-hyperproliferative activity. Conventional formulation of MF has various limitations, such as poor uptake through the stratum corneum, swelling of the hair follicle, and skin burning after prolonged use. Further, its systemic absorption leads to various adverse effects, such as reversible suppression of Cushing’s syndrome, hyperglycemia, glycosuria, etc. [46] To minimize these systemic and local side effects and enhance the retention of the drug at the target site, Kaur et al. developed MF-loaded NLCs. The MF-NLCs were formulated by a microemulsion technique. The optimized MF-NLCs showed PS ~163 nm, ZP −0.086 mV, and EE 60%. TEM studies showed spherical-shaped morphology of the MF-NLCs. Further, the MF-NLC dispersion was incorporated into carbopol 940 for enabling its topical administration. In drug permeation studies, the MF-NLC hydrogel depicted extended drug release as compared to the marketed product, and it followed Higuchi release kinetics. The MF-NLC hydrogel exhibited ~2.5-fold greater skin deposition of MF than that of the marketed product.

In the Draize test, the MF-NLC hydrogel showed slight irritation in comparison to the marketed product after repetitive application over 14 days. In histopathological studies of the psoriasis-induced mouse skin, the MF-NLC hydrogel-treated group showed a healthy keratin layer and an improvement in the thickness of the epidermis. A substantial decrease in hyperkeratosis, parakeratosis, and hyperplasia was noticed after 14 days of application of the MF-NLC hydrogel.

Further, in storage stability studies, the MF-NLC hydrogel was found to be stable over a one-month study period [46].

### 5.4. Patent Information

Plentiful research work on NLC-based formulations has been carried out in the last few years and patented under various patenting agencies. Here, we have performed a specific patent search on the World Intellectual Property Organization (WIPO) and Google patent search databases and shortlisted the seven most relevant patents published on NLC-based formulations for the treatment of psoriasis. Relevant details on these patents are summarized in Table 3 [47,48,49,50,51,52,53].

## 6. Regulatory Aspects

For regulation and control of NLC-based nanoformulations, there is no unanimous guideline that exists. However, important regulatory agencies such as the FDA, the MHRA, etc. prefer to assess the nanoformulations on a case-by-case basis, i.e., in a product-specific way. For regulating nanoformulations in India, “the guidelines for evaluation of nanopharmaceuticals in India” were prepared by the Department of Biotechnology in collaboration with the Indian Society of Nanomedicine. Further, a three-tiered framework has been developed for assisting the governing bodies to regulate nanomedicines. In the USA, the USFDA established a nanotechnology task force and a nanotechnology interest group to address regulatory concerns worldwide. The Nanotechnology Characterization Laboratory of the National Cancer Institute (NCL-NCI) has played an important role in assessing the nanomedicines. In the UK, the Medicines and Healthcare Products Regulatory Authority (MHRA) governs the nanomedicines. In the UK and Europe, the European Nanomedicine Characterization Laboratory (EU-NCL) provides information on preclinical assays of nanomedicines. In Europe, initiatives such as the EU-NCL, the REFINE project, and preliminary guidelines developed for consultation by the European Medicines Union (EMU) have played a vital role in this field. In Canada, Health Canada, the Health Portfolio Nanotechnology Working Group, and the Canadian Institutes of Health Research (CIHR) are responsible for discussing and governing the issues related to nanomedicines [54,55].

It is a well-accepted fact that to safeguard, promote, and preserve public health, regulatory authorities must constantly monitor any formulations. When it comes to polymeric nanoparticles, the excipients employed occasionally do not fall within the GRAS category, because the FDA has not given them regulatory approval. They might produce positive experimental findings, but they cannot be applied to commercial products. The development of a new drug delivery method should not be impeded, and regulatory agencies should make sure that pharmaceutical companies perform a range of toxicity tests on every new medication or new ingredient, such as polymers or lipids, before generating formulations from them. The significance of rules for a new pharmaceutical product has increased as a result of all the emerging nanocarrier systems, including nanoparticles, dendrimers, carbon nanotubes, liquid crystals, and others. The regulatory authorities consider all of the constituents, components, and constituents of NLCs, including lipids and emulsifiers, to be physiologically safe, nontoxic, invulnerable, biodegradable, and biocompatible in nature, and they easily fall into the GRAS category. Nevertheless, it is crucial to employ all the substances within safe and acceptable limits. The majority of them are made from substances that are found in the human body naturally, such as fatty acids and glycerol. It has also been observed that they are currently being utilized in food products as well as, in some cases, for the safe and acceptable encapsulation of medicinal substances. The widespread acceptance and commercial success of NLCs are largely due to minor regulatory challenges. Few NLC-containing products have acquired regulatory approval and are readily available on the market [56,57].

## 7. Summary

The successful treatment of psoriasis is still of major concern due to the increasing proportion of patients and the lack of appropriate treatment options. The extensive literature reports available illustrating various examples bring to our attention the focus on NLCs as a transdermal delivery system for anti-psoriatic drugs. These novel physiochemically stable systems could be a boon in enhancing penetrability, retention, bioavailability, biosafety, occlusive properties, sustainability, and many such desirable properties. They are robust and effective carriers for lipophilic molecules in particular, but they also have the ability to entrap both lipophilic and hydrophilic medicines and to increase skin hydration, elasticity, and also dispersibility in an aqueous media. In this review, the various case studies demonstrate the successful outcomes established by the development of NLCs as anti-psoriatic drugs. NLCs seem to have more industrial applications due to their scale-up feasibility, lipids being GRAS approved, and the possibility of large-scale production. In this review, we conclude that the psoriasis site-specific targeting of the medicine through dermal administration is promising, with little systemic availability and minimal adverse effects using NLCs.

After performing an overview of policies implemented across the globe to regulate nanoformulations, it was observed that there are inconsistencies in regulating nanopharmaceuticals among different government agencies.

## 8. Future Prospective

The current review has emphasized the recent advances in the delivery of anti-psoriatic drugs through NLCs. Their numerous advantages over first-generation systems make us consider this novel drug delivery system as the future best alternative for the treatment of psoriasis. This delivery system is deemed to pave a new way for lipid-based carriers. However, despite the extensive effort in the generation of NLCs and preclinical investigations, enough clinical studies have not been conducted to ensure the therapeutic value of innovative NLCs in psoriasis, which demonstrates the necessity for more clinical and safety research in this area. The only remaining concern about the commercial fate of these NLCs for anti-psoriatic therapy is the risk of any unpredicted health hazards due to these nanomaterials. Further, in the future, it is expected that various regulatory agencies such as the FDA, the MHRA, CDSCO, etc. should work collaboratively and create collective and stringent guidelines for the production and control of nanoformulations. Therefore, in nutshell, it can be inferred that it is possible to achieve success in the utilization of NLCs for psoriasis if the preliminary academic research is confirmed by industry experts and regulatory agencies.

## Figures and Tables

**Table 1:** Present therapies for the treatment of psoriasis and their limitations.

Sr. No.	Type of Therapy	Drug Candidates	Adverse Effects of Current Therapy
1.	Topical therapy (ointments, creams, lotions, gels, or foams applied to the skin	Vitamin D3 analogues (e.g., calcitriol, calcipotriol)	Cutaneous irritation, itching, burning, dryness of skin
		Corticosteroids, e.g., betamethasone dipropionate, hydrocortisone, clobetasol propionate, triamcinolone acetonide, mometasone furoate, Fluocinolone acetonide, etc.	Dermal atrophy, acne-like skin, decreased pigmentation, allergic contact dermatitis, and their long-term use suppresses the body’s immune system
		Anthralin/dithranol	Irritating, burning, staining, and necrosis
		Retinoids, e.g., tazarotine, acetratin, etretinate	Cheilitis, alopecia, desquamation, drying of mucous membranes, pruritus, etc.
		Calcineurin inhibitors, e.g., tacrolimus, pimecrolimus	Burning, pruritus, and erythema
		Tar	Irritation, rash, or acne-like skin, skin burning, and makes skin more subtle to ultraviolet (UV) light
		Diathranol	Skin irritation, pruritus, erythema, dry skin, eczema rash, etc.
		Keratolytic agents, e.g., omega-3 fatty acids, cod liver oil, salicylic acid, etc.	Skin irritation, burning, peeling, etc.
2.	Phototherapy	UV-B therapy Psoralen plus UV-A therapy (PUVA)	Photo aging, sunburn, and erythema
3.	Systemic therapies (tablets or injections/infusion)	Methotrexate	Nausea, malaise, elevated transaminases, bone marrow suppression, gastrointestinal ulcers, pneumonitis, etc.
		Cyclosporin	Kidney failure, hypertension, gingival hyperplasia, headache, hypertrichosis
		Acitretin	Hyperlipidemia, liver toxicity, teratogenicity, mucocutaneous lesions, etc.
		Biologic agents, e.g., infliximab, etanercept, adalimumab, secukinumab, etc.	Candidiasis observed with secukinumab in initial stages of therapy; infusion reactions common with infliximab
		Oral small molecules, e.g., apremilast	Diarrhea, augmented gastrocolic reflex, depression, weight loss, upper respiratory tract infection (URTI), nasopharyngitis, etc.

**Table 2:** Various solid lipids, liquid lipids, surfactants, other excipients, and solvents are used in the fabrication of NLCs in psoriasis treatment.

Sr. No.	Categories of Excipients					In Vitro/Ex Vivo Studies	In Vivo Studies	Ref.
	Solid Lipids	Liquid Lipids	Surfactants/Co-Surfactants (Emulsifier)	Other Excipients	Organic Solvents			
1.	Palmitic acid	Oleic acid	Surfactant mixture (smix) of Tween 20^®®^: Span 60^®®^: n-butanol in a ratio 3.5:1.0:0.5	-	-	NLCs exhibited initial sustained release phase for the first 10 h and subsequent steady state release of Acitretin.	Clinical study depicted significant reduction in erythema, followed by marked reduction in scaling, indicating moderate to excellent improvement in the disease symptoms.	[4]
2.	Glyceryl distearate (Precirol^®®^ ATO 5)	Oleoyl polyoxyl-6 glycerides (Labrafil M1944 CS)	Poloxamers and polysorbate 80	-	-	Curcumin-NLCs demonstrated 48 h in vitro release with 3.24-fold improved permeation and skin retention.	-	[5]
3.	Compritol^®®^ 888 ATO	Medium chain triglyceride (MCT) (Miglyol^®®^ 812)	Polysorbate 80	-	Methanol and acetone (1:1, *v*/*v*): for dissolving lipids and drug	Fluocinolone acetonide NCLs showed prolonged drug release with significant amount of drug in epidermal and dermal layer of skin.	-	[18]
4.	Compritol 888	Capmul^®®^ MCM	-	-	Acidified isopropyl alcohol (IPA): to dissolve lipids	Methotrexate release with sustained and steady release over a period of 30 h	Methotrexate NLC gel exhibited significant anti-psoriasis activity as compared to the control and plain methotrexate gel formulation.	[20]
5.	Compritol 888 ATO	Oleic acid	L- α egg phosphatidylcholine	-	Acetone and ethanol(1:1): to dissolve drug	Capsaicin NLCs showed better drug penetration than SLNs with higher amount of drug retention.	Capsaicin NLCs showed 8.7-fold higher accumulation of drug in viable skin.	[29]
6.	Glyceryl monostearate	Castor oil (Cremophor RH40)	Sorbitan monolaurate (Span 20) PEG-40 Hydrogenated	Carbopol^®®^ 934P NF polyacrylic acid-based gelling agent: for better hydration and retention of gel	-	HaCaT cell line study revealed higher uptake and efficacy due to decreased cell viability for NLC.	The anti-psoriatic effect increased significantly as compared to drug-loaded SLN and marketed formulations.	[30]
7.	Combinations of hard fat, polyoxyethylene, (25) cetyl stearyl ether, and glyceryl ricinoleate (stabilized) (Witepsol^®®^ S51);	Oleic acid	Polysorbate 60 and polysorbate 80	-	-	Methotrexate was released from the NLCs in vitro in a fast release pattern, reaching 70% in 2 h. Increased skin permeation on methotrexate compared with the free drug.	-	[31]
8.	Glyceryl palmitostearate (Precirol) Palmitinic acid monoglycerides (Myverol™ 18–04 K)	Squalene	Polyoxyethylene-polyoxypropylene block copolymer (pluronic F-68)	-	Chloroform: methanol (2:1 *v*/*v*): for dissolving lipid	In vitro permeation study suggests calcipotriol NLCs easily penetrated into the stratum corneum. Skin permeation of methotrexate NLCs was enhanced 2.4 to 4.4 times versus control.	Confocal laser scanning microscopy (CLSM) showed good topical delivery of calcipotriol and methotrexate.	[34]
9.	Glyceryl dibehenate (Compritol^®®^ 888 ATO)	Isopropyl myristate	Tween 80	Butylated hydroxytoluene (BHT): preservative	Ethanol: to dissolve tretinoin drug and BHT	Tretinoin NLCs provided significantly higher skin retention and permeation when compared to other products such as liposomes, ethosomes, SLNs, etc.	The mouse tail model demonstrated improved tretinoin NLC efficacy. NLCs demonstrated comparable and improved orthokeratosis compared to other drug products.	[37]
10.	Compritol^®®^ 888 ATO	Oleic acid	Polysorbate (Tween) 80 or 60 or 20	Carbomers (Carbopol^®®^ 974P NF, Carbopol^®®^ 980, Carbopol^®®^ 940): for preparation of NLC-gel	Transcutol P: as a penetration enhancer	Apremilast NLCs showed sustained release over a period of 9 h. Apremilast NLC-based hydrogel showed 60.1% skin deposition.	-	[38]
11.	Glyceryl monostearate	Oleic acid	-	Carbomer 934: as geling agent	Acetone and ethanol (1:1 *v*/*v*): for dissolving lipids with/without drug	Methotrexate-loaded NLC hydrogel showed prolonged release over period of 24 h.	-	[39]
12.	Glycerol distearate	Oleic acid	Poloxamer 407	Polyethylenimine (PEI): a transfection agent for siRNA	-	Tacrolimus NLCs showed sustained release over a period of 24 h with zero-order release kinetics.	NLCs prevented the appearance of inflammation and psoriatic plaque with significant inhibition of TNF-α.	[40]
13.	Precirol^®®^ ATO 5	Triglycerides of caprylic acid and capric acid (Captex^®®^ 300);	Poloxamer 407	Mannitol: cryo protectant in freeze drying	-	Methotrexate NLCs exhibited sustained release over a period of 48 h.	Imiquimod-induced psoriasis model depicted normal skin structure with very mild keratosis.	[41]
14.	Precirol^®®^ ATO 5	Glyceryl monocaprylate (Capmul^®®^ MCM C-8 EP)	Tween 80^®®^	Triethanolamine: to maintain the pH of NLC-nano gel	Methanol: to dissolve curcumin drug	Curcumin NLCs depicted sustained release over the period of 24 h (in vivo) and prolonged permeation for 24 h (ex vivo).	-	[42]
15.	Precirol^®®^ ATO 5	Propylene glycol dicaprolate (Labrafac™ PG)	Tween 20^®®^	Methylcellulose: thickening/suspending agent in preparation of NLC-nanogel	Chloroform: to dissolve drug and antioxidants	Dithranol NLCs exhibited sustained release of dithranol over 24 h.	Imiquimo-induced psoriatic plaque model suggests reduction in psoriatic symptoms in mice.	[43]
16.	Butyrospermum parkii (Shea) butter (Jarplex™ SB35)	-		-	Acetone: to dissolve lipids and drug	In contrast to normal skin, psoriatic skin depicted higher levels of curcumin deposition in the epidermis.	Alleviation of psoriatic skin symptoms in imiquimo-induced mice	[44]
17.	Bacuri butter	Tucuma oil	Sorbitan monooleate (Span 80^®®^) and polysorbate 80 Chitosan: to coat NLCs for enhancing bio adhesion and improving biopharmaceutical properties	-	-	NLCs depicted improved cellular uptake, cell viability, and fibrolast uptake of fucoxanthin.	-	[45]
18.	Stearic acid	-	Tween 80^®®^ and Transcutol P (2:1 ratio)	-	-	Permeation study revealed prolonged release of mometasone furoate with Higuchi release kinetics.	Treatment with the prepared NLC formulation completely eliminated the parakeratosis in the imiquimo-induced mouse model.	[46]

**Table 3:** Patents relevant to NLCs revealing their therapeutic potential against psoriasis.

Sr. No.	Title of Patent	Name of Inventors	Patent Grant No./Application No.	Summary of Invention
1.	A Method of Preparation of Triamcinolone Acetonide-Encapsulated Nanostructured Lipid Carriers For Psoriasis Treatment	Pradhan, M; Sahu, K.K.; Singh, D.; Singh, M.R.; Yadav, K.	AU2021106678	Triamcinolone acetonide-loaded NLCs (TA-NLCs) were fabricated by melt dispersion technique for the treatment of psoriasis. Docosahexaenoic acid (DHA) was incorporated to serve a dual role as an excipient as well as offer anti-inflammatory activity. Omega-3 fatty acid was used to enhance transepidermal delivery. Further, the NLCs were incorporated into the hydrogel matrix (TA-NLC hydrogel) for ease of application, proper spreading, and longer contact time. The optimized TA-NLCs showed PS, PDI, ZP, EE, and DL of 168.9 nm, 0.247, −26.6 mV, 81.12%, and 21.61%, respectively. TA-NLCs and hydrogels followed the Korsmeyer Peppas and Higuchi kinetic model with R2 values of 0.9528 and 0.9063 respectively.
2.	Nanostructured Lipid Carriers Containing Tazarotene and Pharmaceutical Formulations Containing Said Particles	Parmar, M.; Patel, L.D.; Rathod, L.; Parikh, K.	IN201921023616	Tazarotene-loaded NLCs (TAZ-NLCs) were formulated by melt emulsification and probe sonication methods. Cutina GMS, Cremophor EL, polyvinyl alcohol, and water were used as the solid lipid, liquid lipid, surfactant, and water, respectively. TAZ-NLCs exhibited an average diameter of nearly 160–250 nm and a ZP ±30 mV. TAZ-NLCs were further added in carbopol gel for topical application.
3.	Antipsoriatic Effects of Clobetasol-Loaded Nanostructured Lipid Carriers on Imiquimod-Induced Psoriasis	Kudamala, R.R.; Suggala, V. S.; Veeram, J. R.; Palagati, S.	IN202141009486	Clobetasol-17-propionate-loaded NLCs (CLB-NLCs) were formulated by melt emulsification and ultra-sonication technique lyophilized using 2% w/v mannose as a cryoprotectant. CLB-NLCs with lipid: drug ratio of 7.5:1 and surfactant (Tween 20) at 1.5% w/v showed lesser PS, PDI, and greater EE of 81.3%. CLB-NLCs were further poured into a gel using gel 1% *w*/*w* carbopol 934. The ointment was formulated by melt dispersion method by melting paraffin wax and stearyl alcohol under heating, followed by the addition of BHT and clobetasol. Percent release of CLB from ointment and gel system was found to be slower, i.e., 35.1% and 74.6%, respectively after 24 h as compared to its solution form, which showed faster release of 94.5% within 7 h. Epidermal thickness was greatly reduced in CLB-NLC gel-treated mice as compared to IMQ-treated mice. CP-loaded NLCs gel showed a greater decline of IL-17, IL-22, and IL-23 levels i.e., 52.9, 72.2, and 74.9% respectively as compared to ointment and negative control, and positive control groups. TNF-α levels were found to be decreased by 51.2, 36.8, and 49.2% in CLB-NLCs, ointment, and positive control treated groups respectively.
4.	Clobetasol-Loaded Solid Lipid Nanoparticles and Nanostructured Lipid Carriers for Topical Treatment of Psoriasis	Kudamala, R.R.; Shaik, C. B.; Veeram, J.R.; Medarametla, K. B.; Anna, B.; Challa M.C.; Chiruthanur, G.; Ponnaiah, B.R.K.; Ranganatham, V.P.; Palagati, S.	IN202141046636	Clobetasol-loaded SLNs were formulated by emulsification-homogenization method for the treatment of psoriasis. A drug–lipid (Compritol) ratio of 1:4 and surfactant (Tween 80 (3%)) were used for the formulation of SLNs. CLB-loaded NLCs were formulated by emulsification and sonication methods. CLB-NLCs with drug lipid ratio (CLB to Compritol and oleic acid) of 1:7.5 showed significantly lower PS (even lower than SLNs). Sodium azide (0.02%) was added to NLCs to prevent microbial growth. At lower drug loading, NLCs displayed faster drug release than SNLs. At high drug loading, no noteworthy difference in drug release from SLNs and NLCs was observed. The study demonstrated CLB-loaded NLC gel had higher efficacy compared to SLNs and marketed formulations in psoriatic management.
5.	Topical Composition	Shah, M.; Panigrahi, L.; Patravale, V.; Kakade, P.	IN201921019828	Apremilast or its pharmaceutically acceptable salt, ester, or prodrug was formulated using a li composition in various forms such as NLCs, SLNs, liposomes, niosomes, ethosomes, transferosomes, etc. NLCs consisted of solid lipid, liquid lipid, surfactant, co-surfactant, and water. Development of NLCs involved the following steps: (A) Drug in the co-surfactant system: drug was solubilized in an optimized co-surfactant system. (B) Lipid phase: solid lipid and liquid lipid were added in the above system and melted in a water bath at nearly 60 °C. (C) Aqueous surfactant phase: surfactant was added in pre-heated water at 50–80 °C. (D) Emulsification step: lipid and aqueous surfactant phases were mixed using a cyclomixer or overhead stirrer at a speed of ~1000 rpm to obtain microemulsion; that was further dispersed in water under stirring at ~3000 rpm.
6.	Tripterine Nanostructure Lipid Carrier and Preparation Method and Application Thereof	Yan, C.; Zhenhai, Z.; Zhou, Lei; Qingqing, Wu.	CN102225205B	Tripterine-NLCs were prepared containing 1 part (by weight) of tripterine, mixed lipid 5–100 parts, phospholipid 0.5–10 parts, poloxamer-188 0.1–15 parts, and vitamin E and tocopherol polyethylene glycol succinate (TPGS) 0.5–10 parts. Lipid phase consisted of monoglyceride solid lipid and octylic acid/caprin liquid lipid in a 1: 0.1–10: 1 weight ratio. Tripterine-NLCs were applied by transdermal route in semi-solid or liquid dosage forms such as ointment, gel, emplastrum, or spray. Tripterine-NLCs depicted particle diameter of 95–150 nm, ZP -20 mv–-50 mv, EE 89–98%, drug loading 8.2–12%, and longer stability after the lyophilization. Tripterine-NLCs could be responsible for improving and enhancing the safety, improving the bioavailability, and reducing the first-pass metabolism of tripterine.
7.	Novel Nanoparticle Formulations for Skin Delivery	Sachdeva, M.S.; Patlolla, R.	US20120195957A1	DID–oil or DIO fluorescent dye-loaded NLCs (DID-NLCs or DIO-NLCs) and Celecoxib (CLX)-loaded NLCs were fabricated by hot melt homogenization technique, and their surface was modified by cell-penetrating peptides (CPPs). DID-NLCs or DIO-NLCs showed PS 160 ± 20 (~173) nm and ZP −11.7 mV, whereas CLX-NLCs showed PS ~167 nm, ZP −25.01 mV, and EE 98%. CPP surface-modified peptides showed enhanced permeation of CLX through the skin as compared to plain NLCs and control peptide YKA-coated NLCs.

## Data Availability

Not applicable.
